# SDF-1 loaded dECM patch improves cardiac function in rats after myocardial ischemia

**DOI:** 10.1186/s13287-026-05188-x

**Published:** 2026-07-18

**Authors:** Jing Zhang, Wanqing Lin, Qian Li, Shiyu Cui, Xiang Bu, Xin Jiang, Aiqun Ma, Tingzhong Wang

**Affiliations:** 1 Department of Cardiology, Shaanxi Provincial People’s Hospital, Xi’an, 710068 Shaanxi China; 2 Laboratory for Multiscale Mechanics and Medical Science, Xi’an Jiaotong University, Xi’an, 710049 Shaanxi China; 3 Department of Cardiology, The First Affiliated Hospital of Xi’an Jiaotong University, Xi’an, 710061 Shaanxi China

**Keywords:** Myocardial infarction, Bone marrow mesenchymal stem cells homing, Stromal cell-derived factor-1, Wall stress

## Abstract

**Background:**

Ischemic heart disease (IHD) remains a global health challenge, characterized by irreversible cardiomyocyte loss and pathological ventricular remodeling. Myocardial patches provide mechanical support for infarcted myocardium, but conventional biomaterials lack bioactive components to facilitate tissue regeneration. This study introduces a novel therapeutic strategy: a stromal cell-derived factor-1 (SDF-1)-loaded decellularized extracellular matrix (dECM) (SDF-dECM) patch addressing both biological and biomechanical deficiencies in myocardial repair.

**Methods:**

Porcine left ventricular dECM was decellularized, and SDF-1 was loaded to fabricate the SDF-dECM patch. The patch’s physicochemical properties, biocompatibility, and SDF-1 release profile were evaluated via hematoxylin and eosin/4’,6-diamidino-2-phenylindole staining, scanning electron microscopy, cytotoxicity assay, and enzyme-linked immunosorbent assay (ELISA). Transwell assays determined the chemotactic effect of SDF-1 on bone marrow mesenchymal stem cells (BMMSCs) and optimal concentration. Uniaxial tensile testing assessed mechanical properties. A finite element method (FEM) model was established to evaluate the patch’s mechanical support. In vivo, male Sprague-Dawley rats were randomly divided into four groups (*n* = 15 per group): Sham, myocardial infarction (MI), MI + dECM, and MI + SDF-dECM. Echocardiography, histochemical staining, and immunofluorescence staining were used to assess cardiac function, tissue morphology, BMMSCs homing, microvascular regeneration, and cardiomyocyte apoptosis. The paracrine mechanism of BMMSCs was evaluated via ELISA and functional assays.

**Results:**

The SDF-dECM patch achieved successful decellularization, maintained a porous structure (50-150 μm pore size), exhibited favorable biphasic mechanical properties, and exhibited sustained SDF-1 release for 28 days. SDF-1 effectively induced BMMSCs migration via the SDF-1/CXCR4 axis, with 200 ng/mL as the optimal concentration. In vivo, the SDF-dECM patch significantly enhanced endogenous BMMSCs homing to the peri-infarct area, promoted peri-infarct microvascular regeneration, and inhibited cardiomyocyte apoptosis. BMMSCs-conditioned medium contained high levels of vascular endothelial growth factor and hepatocyte growth factor, exerting pro-angiogenic and anti-apoptotic effects. FEM simulations showed the patch reduced infarct zone wall stress and stress concentration. Echocardiography and histological analysis confirmed the SDF-dECM patch significantly improved cardiac function, reduced infarct size and alleviated myocardial fibrosis.

**Conclusion:**

The dual-functional SDF-dECM patch integrates sustained SDF-1 delivery for endogenous BMMSCs mobilization and biomechanical support to mitigate ventricular remodeling. The synergy between biological activity and mechanical stabilization provides a promising therapeutic strategy for IHD, overcoming limitations of conventional biomaterials.

**Supplementary Information:**

The online version contains supplementary material available at https://doi.org/10.1186/s13287-026-05188-x.

## Introduction

Ischemic heart disease (IHD), characterized by coronary ischemia and subsequent myocardial injury, is a major global health burden and a leading cause of mortality worldwide [1, 2]. Due to the limited regenerative capacity of cardiomyocytes [3], IHD induces cardiomyocyte apoptosis and necrosis, leading to myocardial fibrosis, scar formation, progressive left ventricular remodeling, and ultimately heart failure (HF) [4]. Current pharmacological and surgical interventions cannot repair severely damaged or necrotic myocardium, resulting in persistently high mortality and highlighting an urgent need for more effective strategies.

Tissue engineering is a promising strategy for enhancing cardiac function by integrating stem cells, cytokines, and scaffolds to repair damaged myocardium [5]. Early pioneering work by Badylak et al. demonstrated the potential of extracellular matrix (ECM) as an inductive scaffold for myocardial replacement [6], laying the foundation for decellularized extracellular matrix (dECM)-based cardiac repair. From a mechanical standpoint, myocardial infarction (MI) causes left ventricular wall thinning, elevating wall stress (WS) and accelerating ventricular remodeling, which progresses to HF [7]. According to Laplace’s law [8], myocardial patches mitigate ventricular remodeling by increasing wall thickness, sharing mechanical load, and reducing excessive diastolic deformation. Among patch materials, dECM patches are particularly promising due to their excellent biocompatibility, natural three-dimensional structure [9], and capacity to support cell adhesion and survival [10]. Both non-myocardial dECM [6, 11] and porcine myocardial dECM [12, 13] have shown promise in cardiac repair, with their robust mechanical properties providing critical support to infarcted tissue and alleviating ventricular remodeling [14, 15]. Notably, myocardial dECM possesses inherent bioactivity, promoting post-MI constructive remodeling via enhanced angiogenesis and progenitor cell recruitment (correlating with functional restoration) [12, 13], regulating mesenchymal stem cells (MSCs) phenotype, and improving mechanical properties post-cell seeding in vitro [16, 17]. However, dECM patches alone lack sufficient efficacy in driving robust cardiomyocyte regeneration, prompting exploration of bioactive factor incorporation to enhance their regenerative potential.

Cell therapy is another critical approach for severe myocardial ischemia [18], but early studies identified challenges including poor transplanted cell retention and survival (over 90% die within 24 h post-transplantation [19]), high myocardial contraction pressure, uneven cell distribution [20], and injection-related mechanical damage. Endogenous stem cell therapy has thus become a more viable post-MI cardiac repair strategy [21]. Stromal cell-derived factor-1 (SDF-1) is upregulated post-acute MI; it binds to C-X-C chemokine receptor type 4 (CXCR4) on endogenous stem cells, facilitating their migration to the injury site and promoting myocardial repair via paracrine signaling [22, 23]. SDF-1 activation improves post-MI ventricular function by inducing neovasculogenesis [24], and controlled SDF-1 release enhances cardiac function via endogenous stem cell recruitment [25–27]. However, SDF-1 upregulation is transient [28], and local exogenous SDF-1 injection yields short-lived effects [29–31], emphasizing the need for a sustained delivery system. The dECM, with its natural three-dimensional porous structure, serves as an ideal platform for sustained SDF-1 release and long-term stem cell survival [32].

**Fig. 1:**
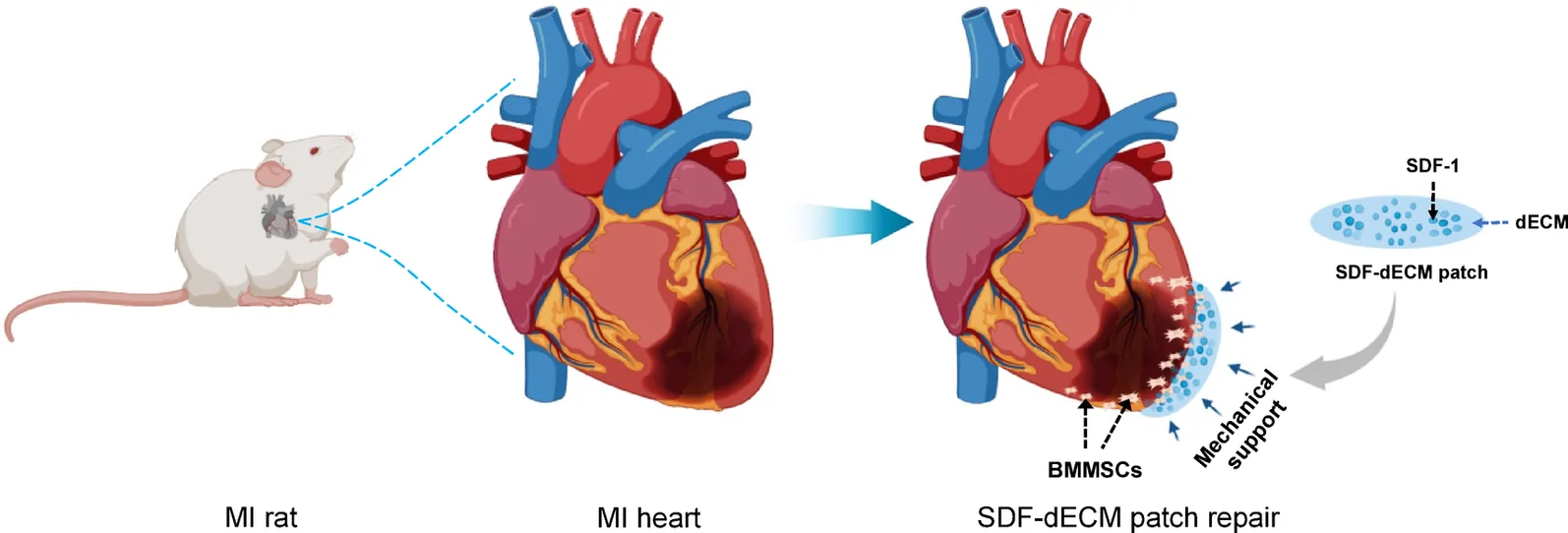
Roadmap of SDF-dECM patch implantation. A schematic showing the process of implanting the SDF-dECM patch onto infarcted myocardium

In this study, we developed an SDF-1-loaded dECM (SDF-dECM) patch that integrated bone marrow mesenchymal stem cells (BMMSCs) homing and mechanical support to improve cardiac function after MI in rats (Fig. 1). This design leveraged the unique advantages of myocardial dECM over synthetic biomaterials: its inherent bioactivity promoted constructive tissue remodeling [12, 13], and its tissue-specific composition and three-dimensional architecture provided a biomimetic microenvironment for BMMSCs recruitment and retention [33]. The SDF-dECM patch exerted a dual therapeutic effect: (1) sustained release of SDF-1 to promote endogenous BMMSCs homing to the infarcted region, and (2) mechanical support to reduce WS and inhibit ventricular remodeling. This integration of biological signaling and biomechanical support represented a promising strategy for the treatment of IHD.

## Methods

### In vitro study design

In vitro experiments were designed to fabricate and characterize the SDF-dECM patch, as well as to evaluate its biocompatibility, SDF-1 release kinetics, and bioactivity. Experiments were performed sequentially following this workflow: (1) Patch fabrication and characterization via histological staining including hematoxylin and eosin (H&E) and 4’,6-diamidino-2-phenylindole (DAPI), DNA quantification, and scanning electron microscopy (SEM); (2) Biocompatibility assessment using Cell Counting Kit-8 (CCK-8) assay and live/dead staining, with rat BMMSCs and H9c2 cardiomyocytes exposed to patch extracts; (3) Quantification of SDF-1 release kinetics over a 28-day period using enzyme-linked immunosorbent assay (ELISA); (4) Functional evaluation of the released SDF-1 via Transwell migration assay and tube formation assay. 

### In vivo study design

The in vivo study was designed to investigate the therapeutic efficacy of the SDF-dECM patch in a rat model of MI. Male Sprague-Dawley rats (*n* = 15 per group) were randomly assigned to four groups: Sham, MI, MI + dECM, and MI + SDF-dECM with the detailed modeling procedure described later. The primary endpoint was the change in cardiac function at 4 weeks post-surgery, assessed by echocardiography. Secondary endpoints included infarct size (evaluated via Masson Trichrome staining), capillary density (assessed by CD31 immunofluorescence), cardiomyocyte apoptosis (detected by TUNEL assay), stem cell homing (identified via CD44/CD29/CD90/CXCR4 immunofluorescence), and host immune response (evaluated by CD3/CD68 immunofluorescence). These endpoints were analyzed using heart tissue harvested at 2 and 4 weeks post-surgery. Additionally, the work has been reported in line with the ARRIVE guidelines 2.0.

### Materials

BMMSCs (RRID: CVCL_6D69) were isolated as primary cells from rat bone marrow. H9c2 cardiomyocytes (Cat# CL-0089, RRID: CVCL_0286, Wuhan Procell Company) and cardiac microvascular endothelial cells (CMECs) (Cat# CP-R135, RRID: CVCL_4312, Wuhan Procell Company) were purchased from Wuhan Procell Company. Male Sprague-Dawley rats (6-7 weeks old, weighing 200-220 g) were obtained from the Laboratory Animal Center of Xi’an Jiaotong University. All rats were housed in a controlled environment (temperature 22 ± 2 °C; 12 h light/dark cycle) with free access to food and water. Rats were anesthetized with intraperitoneal sodium pentobarbital (50 mg/kg) for cardiac surgery. Following echocardiography and tissue collection, all surviving rats were humanely euthanized by an overdose of intraperitoneal sodium pentobarbital. All experimental procedures were reviewed and approved by The Biomedical Ethics Committee of Health Science Center of Xi’an Jiaotong University (Project Title: Study on the effect and mechanism of myocardial ischemia-induced arrhythmia; Approval No.: 2018-163; Approval Date: Feb 23, 2018).

### Fabrication and characterization of porcine myocardial dECM

Porcine myocardial dECM was prepared from porcine left ventricles using a standardized decellularization protocol [10]. Briefly, porcine hearts were agitated in 1% sodium dodecyl sulfate at 4 °C for 72 h, followed by treatment with 1% Triton X-100 for 24 h. All solutions were replaced every 24 h, and the samples were subsequently rinsed extensively with phosphate-buffered saline for 48 h. All steps were performed under sterile conditions, with 4 biological replicates per group. Successful decellularization was initially verified by histological assessment: 500 μm-thick tissue sections from 4 independent samples per group were stained with H&E and DAPI, then imaged using a confocal microscope (Zeiss LSM 880) under standardized parameters. Thin dECM slices were prepared as previously described [10, 15].

### Preparation of SDF-dECM patches

dECM materials were freeze-dried using a vacuum freeze-dryer for 72 h. The resulting porous dECM scaffolds were cut into circular patches (7 mm in diameter, 500 μm in thickness) and immersed in the SDF-1 (Cat#400-32 A, RRID: SCR_023279, Peprotech) solution (200 ng/mL) at 4 °C for 24 h to allow SDF-1 absorption. The SDF-dECM patches were stored at 4 °C until use.

### Cytotoxicity assay

H9c2 cardiomyocytes and BMMSCs were exposed to extracts of the SDF-dECM patch at different concentrations (0.5, 1, 2, and 4 mg/mL), where the concentration represented the mass of patch material per mL of extraction solvent. After 24-48 h, 10 µL of CCK-8 reagent was added to each well, followed by incubation at 37 °C for 2 h. Absorbance was measured at 450 nm using a microplate reader (BioTek Instruments, USA). Relative cell viability (%) was calculated using the formula: [(As - Ab) / (Ac - Ab)] × 100%, where As = absorbance of the experimental sample, Ab = absorbance of the blank control, and Ac = absorbance of the “cells only” control.

### In vivo experimental groups

A total of 60 adult Sprague-Dawley rats were randomly divided into four experimental groups (*n* = 15 per group). (1) Sham group: underwent thoracotomy without left anterior descending (LAD) coronary artery ligation. (2) MI group: subjected to permanent LAD ligation. Briefly, rats were anesthetized, orally intubated, and mechanically ventilated; a left thoracotomy was performed through the fourth intercostal space to expose the heart, and the LAD coronary artery was ligated 2 mm distal to the junction of the left auricle and pulmonary conus. Successful MI induction was confirmed by ST-segment elevation on electrocardiogram. (3) MI + dECM patch group: received dECM patch implantation, which was sutured to the epicardial surface of the infarct border zone using 6/0 absorbable sutures after LAD ligation. (4) MI + SDF-dECM patch group: received SDF-dECM patch implantation, which was sutured to the epicardial surface of the infarct border zone using 6/0 absorbable sutures after LAD ligation. Animals were euthanized 4 weeks post-surgery for endpoint analysis.

### FEM (finite element method) simulation

A computational model of the left ventricle incorporating electrophysiological and mechanical behaviors was developed in COMSOL Multiphysics. The geometry of the infarcted left ventricle and SDF-dECM patch adhesion were reconstructed from experimental data (Fig. S4). The ventricular wall was divided into three layers with distinct myocardial fiber orientations, and its mechanical behavior was modeled using a fiber-reinforced hyperelastic constitutive relation [34]:

1 $$\:W={W}_{isotropic}+{W}_{fiber}$$

Additionally, myocardial electrophysiological signal propagation was governed by the Aliev-Panfilov equations [35]

2 $$ \zeta C_{m} \frac{\partial }{{\partial t}}\Phi + \nabla \bullet ( - D\nabla \Phi ) + \zeta [I_{{ion}} (\Phi ,F,r_{i} )] = 0 $$

where the ratio of the membrane surface area to volume was denoted by * ζ *, the membrane capacitance is represented by $C_{m}$, the conductivity tensor is denoted by D, and the ionic current per unit area is denoted by $I_{{ion}}$. The deformation gradient of the heart is represented by F. Furthermore, the electrophysiological signal can induce contraction of myocardium fiber and the contraction stress $S_{a}$ was calculated by solving the following differential equation:

3 $$ \frac{{\partial s_{a} }}{{\partial t}} = \varepsilon (V)[k(\Phi - \Phi _{r} ) - s_{a} ] $$

where was the delay function [35]

4 $$ \varepsilon (V) = \varepsilon _{0} + \left( {\varepsilon _{0} - \varepsilon _{1} } \right)\exp ( - \exp ( - \xi (\Phi - \Phi _{t} ))) $$

where *k* = 0.005 MPa/mV, Φ_r_ = -80 mV, *ε*_0_ = 0.1 ms^− 1^, *ε*_1_ = 1 ms^− 1^, *ξ* = 1 mV^− 1^ and Φ_t_ = 0 mV. A periodic sinoatrial node electrical signal was applied to the left ventricle upper surface; Eq. (2) was solved to obtain myocardial electric potential, and Eq. (3) to derive fiber contraction stress. Finally, combining ventricular tissue constitutive relations, kinematics, and large deformation equilibrium equations, left ventricular deformation and WS distribution during diastole and systole were obtained. Material parameters are provided in Table S1. A uniform equibiaxial pre-stretch (λ_pre = 1.0 to 1.2) was applied to the patch pre-implantation, treated as an FEM input parameter to quantify its effects on WS reduction, combined with varying patch thickness and area.

### Mechanical testing of SDF-dECM patches

Uniaxial tension tests were performed to characterize dECM patch mechanical properties. Patch samples (*n* = 4 per group) were hydrated in PBS at 37 °C for 30 min, cut into rectangular strips, and thickness measured at three points using a digital micrometer. Uniaxial tensile tests to failure were conducted on a universal testing system (Instron 5944) with a 10 N load cell, in a 37 °C PBS bath to simulate physiological conditions. A 0.01 N preload was applied to remove slack, followed by elongation at a constant strain rate of 0.1%/s. Force and displacement data were recorded; engineering stress (force/original cross-sectional area) and strain (displacement/original gauge length) were calculated. Ultimate tensile strength, Young’s modulus (linear elastic region), and elongation at break were determined for each sample.

### SDF-1 release kinetics from SDF-dECM patch

SDF-1 concentrations were quantified using commercial ELISA kits (E-EL-R3027, RRID: SCR_023280, Elabscience). Release kinetics were evaluated over 28 days using 7 mm diameter, 500 μm thick patches in PBS (37 °C, pH 7.4), with aliquot sampling without full media replacement. At each time point, 250 µL of release medium was withdrawn and replaced with fresh PBS; aliquots were stored at -80 °C until analysis. Data are presented as mean ± standard error of the mean (SEM) (*n* = 3 independent samples). Assays included measurement, standard, and blank wells (triplicate per sample), performed per kit protocols and repeated three times to calculate average target concentrations.

### Quantification of paracrine factors in BMMSCs-conditioned medium (BMMSC-CM)

Conditioned media were collected from BMMSCs cultured under specific conditions. Vascular endothelial growth factor (VEGF) and hepatocyte growth factor (HGF) concentrations were quantified using commercial ELISA kits (VEGF: E-EL-R2603, RRID: SCR_023282; HGF: E-CL-R0341, RRID: SCR_023281; both Elabscience). Media were collected after 24 h of incubation, centrifuged to remove debris, and stored at -80 °C until analysis. Assays were performed in triplicate using samples from 5 independent biological replicates. 

### Tube formation assay for angiogenesis assessment

CMECs were seeded at 1.5-2.0 × 10⁴ cells/well onto growth factor-reduced Matrigel-coated plates, treated with BMMSC-CM, H9c2-conditioned medium (H9c2-CM) or serum-free control medium. BMMSC-CM was collected from passage 3-5 BMMSCs (80% confluence) after 48-hour serum-free culture. After 2, 4, and 6 h of incubation (37 °C, 5% CO₂), tube formation was imaged using an inverted microscope (×40-100 magnification). Total tube nodes (branching points) and branch length were quantified via ImageJ software with the Angiogenesis Analyzer plugin.

### Establishment of hypoxia-ischemia model in H9c2 cardiomyocytes

Hypoxia-ischemia in H9c2 cardiomyocytes was induced via oxygen-glucose deprivation. Briefly, cells were washed with PBS and cultured in glucose-free Dulbecco’s modified Eagle medium (Gibco, USA), then transferred to a hypoxic chamber (Billups-Rothenberg, USA) (1% O₂, 5% CO₂, 94% N₂) at 37 °C for 24 h. Control groups were maintained in complete high-glucose DMEM under normoxic conditions (21% O₂, 5% CO₂), consistent with established in vitro myocardial ischemic injury models.

### Histological analysis

Rat hearts were harvested at predetermined endpoints, perfused with PBS to remove blood, and fixed in 4% paraformaldehyde at 4 °C for 48 h. Tissues were rinsed in PBS, dehydrated via graded ethanol (70% to 100%), cleared in xylene, infiltrated with molten paraffin, and embedded in paraffin blocks. Serial 5 μm sections were cut using a rotary microtome and mounted on positively charged glass slides. H&E staining was used for general morphological assessment; Masson Trichrome staining evaluated collagen deposition and fibrosis in infarct and border zones, with blue-stained collagen areas quantified to assess fibrosis extent.

### Immunofluorescence staining

Double immunofluorescence staining was used to analyze specific protein localization and expression in myocardial sections. Tissues were embedded and sectioned at 10 μm. Sections were permeabilized with 0.3% Triton X-100 (RRID: AB_2891182, Sigma-Aldrich) in PBS for 15 min and blocked with 5% bovine serum albumin (RRID: AB_2864941, Sigma-Aldrich) in PBS for 1 h at room temperature. Primary antibodies against CXCR4 (1:200, ab124824, RRID: AB_10972276, Abcam), CD29 (1:5000, ab179471, RRID: AB_2877642, Abcam), CD44 (1:1000, ab6124, RRID: AB_305897, Abcam), and CD90 (1:3000, ab92574, RRID: AB_10562401, Abcam) were incubated overnight at 4 °C. After washing, sections were incubated with Alexa Fluor 488-conjugated anti-mouse IgG (1:500, RRID: AB_2534069, Invitrogen) for 1 h at room temperature. Nuclei were counterstained with DAPI (RRID: AB_2336788, Abcam), and images were acquired using a Nikon Eclipse C1 confocal microscope with 60×/1.40 Oil, 40×/1.30 Oil, and 20×/0.75 objectives.

### Statistical analysis

Data were presented as mean ± SEM for normally distributed variables or median ± interquartile range for non-normally distributed variables, with n representing independent biological replicates per group. All statistical analyses were performed using GraphPad Prism 7.0 (GraphPad Software, San Diego, CA). Intergroup comparisons were conducted via one-way analysis of variance, with *p* < 0.05 considered statistically significant.

## Results

### Physicochemical properties of the SDF-dECM patch

**Fig. 2:**
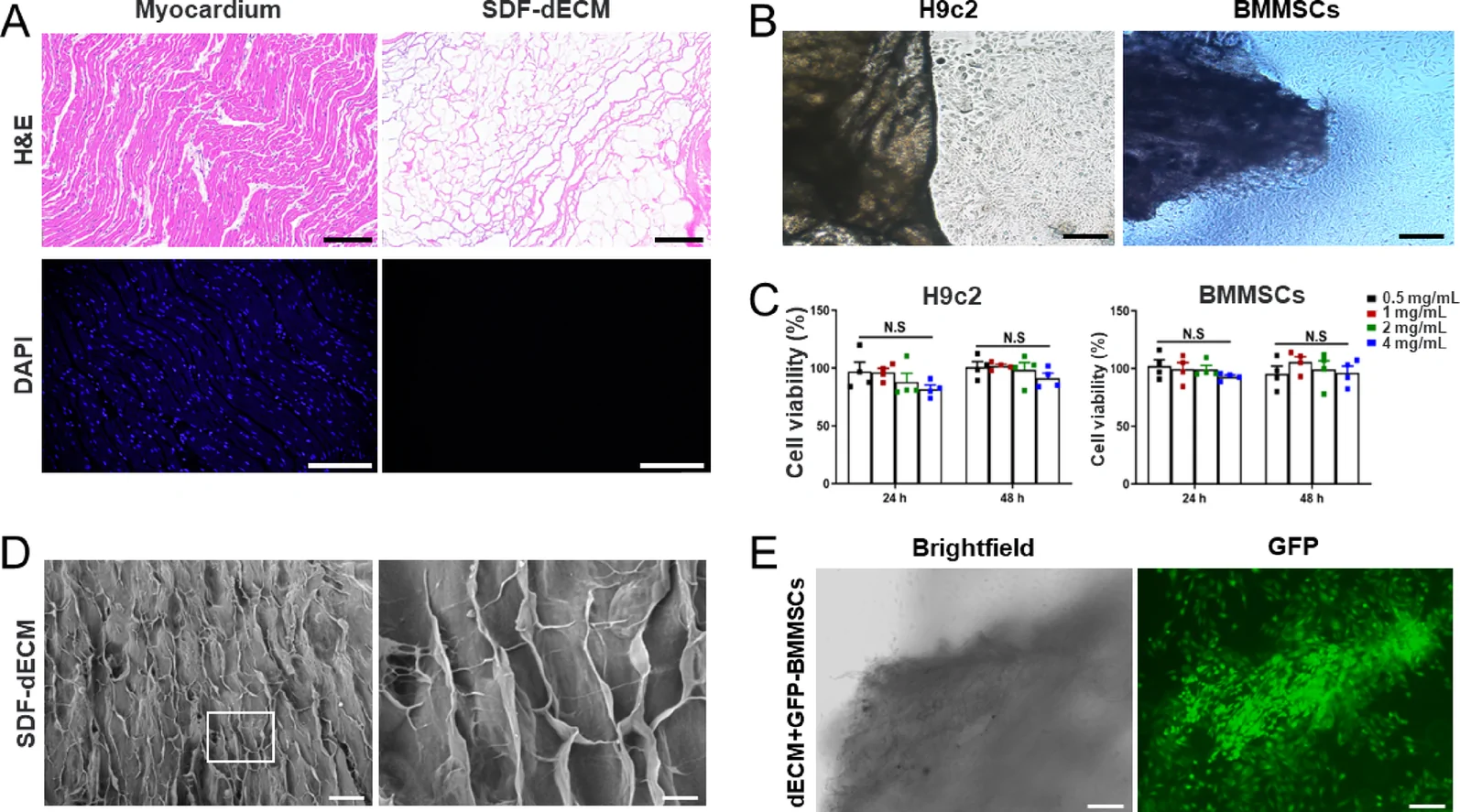
Physicochemical properties of the SDF-dECM patch. **A** H&E and DAPI staining of porcine myocardium (scale bars: 100 μm) and the SDF-dECM patch (scale bars: 50 μm), demonstrating the tissue architecture and cell distribution. **B** Co-culture of the SDF-dECM patch with rat H9c2 cardiomyocytes and rat BMMSCs, respectively. **C** Cell viability of rat H9c2 cells (left) and rat BMMSCs (right) after 24 h and 48 h of exposure to SDF-dECM extracts at various concentrations. N.S. indicates no significant difference. **D** SEM images showing the structural properties of the SDF-dECM patch. Scale bars: 250 μm (left), 50 μm (right). **E** GFP-expressing rat BMMSCs on the SDF-dECM patch were visualized under bright-field (left) and fluorescence microscopy (right) in the same field of view. Scale bars: 100 μm

H&E staining confirmed the removal of cellular components and preservation of native ECM architecture in the SDF-dECM patch compared to native tissue, while DAPI staining showed no fluorescent nuclei across multiple random fields, providing additional qualitative evidence of successful decellularization (Fig. 2A). For biocompatibility assessment, H9c2 cardiomyocytes and BMMSCs were separately cultured with SDF-dECM patch extracts; both cell types displayed robust growth and proliferation around the patch (Fig. 2B), and the CCK-8 assay demonstrated no significant cytotoxicity at patch extract concentrations up to 4 mg/mL (mass of patch material per mL of solvent) (Fig. 2C). SEM revealed a well-preserved, homogeneous porous network in the SDF-dECM patch with an average pore size of 50-150 μm (Fig. 2D). Combined with the absence of nuclei in H&E/DAPI staining and preserved dECM ultrastructure via SEM, successful decellularization was confirmed. Additionally, GFP-expressing BMMSCs showed excellent growth and survival within the patch’s three-dimensional porous structure (Fig. 2E).

### Sustained release of SDF-1 from the SDF-dECM patch

**Fig. 3:**
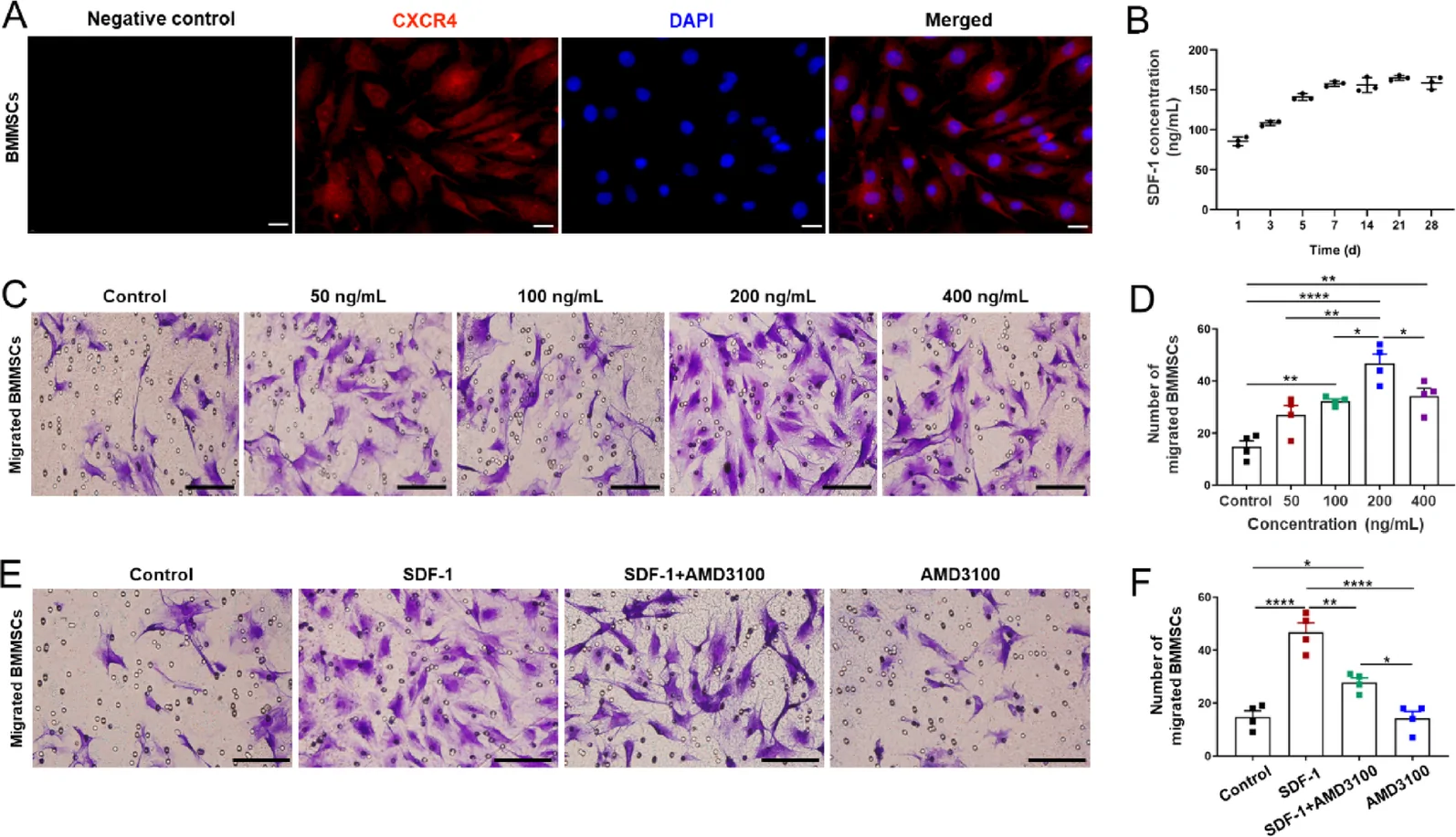
SDF-1 induced rat BMMSCs migration in vitro. **A** Immunofluorescence staining showing CXCR4 (red) expression in rat BMMSCs, with nuclei counterstained using DAPI (blue). Scale bar: 20 μm. **B** In vitro release profile of SDF-1 from SDF-dECM patches over time. Data points represent the concentration measured in the release medium at each time point (mean ± SEM, *n* = 3). Individual data points are shown for each independent sample. **C** Representative images showing rat BMMSCs migration in response to various concentrations of SDF-1. Scale bar: 100 μm. **D** Quantitative analysis of migrated rat BMMSCs across different SDF-1 concentrations. Statistical significance is denoted as *p < 0.05, **p < 0.01, and ****p < 0.0001. **E** Migration of rat BMMSCs in the presence of a CXCR4 inhibitor, AMD3100, showing receptor-dependent migration. Scale bar: 100 μm. **F** Quantitative analysis of rat BMMSCs migration under different AMD3100 conditions. Statistical significance is denoted as *p < 0.05, **p < 0.01, and ****p < 0.0001

Immunofluorescence staining confirmed stable CXCR4 expression on rat BMMSCs (Fig. 3A), indicating their responsiveness to SDF-1, a chemokine that drives BMMSCs migration via CXCR4 binding [36, 37]. ELISA analysis was performed to evaluate SDF-1 release from the SDF-dECM patch; cumulative SDF-1 release was measured over 28 days (*n* = 3). Release increased gradually over the first 7 days, reaching 108.50 ± 1.83 ng/mL by day 3 and peaking at 157.60 ± 1.89 ng/mL by day 7 (mean ± SEM; Fig. 3B), after which the release profile remained stable.

Transwell migration assays were conducted to assess the chemotactic effect of SDF-1 and determine its optimal concentration. SDF-1 at 100, 200, and 400 ng/mL significantly enhanced BMMSCs migration compared to the control (0 ng/mL), with the highest migration observed at 200 ng/mL (identified as the optimal concentration, *p* < 0.05; Fig. 3C, D). To confirm CXCR4’s role in this migration, the CXCR4 inhibitor AMD3100 was used. BMMSCs migration was highest in the SDF-1 group (*p* < 0.0001 vs. control), significantly reduced in the SDF-1 + AMD3100 group (*p* < 0.01 vs. SDF-1 group), and further decreased in the AMD3100-only group (*p* < 0.05 vs. SDF-1 + AMD3100 group) (Fig. 3E, F).

### Mechanical properties of the SDF-dECM patch

Uniaxial tensile testing demonstrated favorable elastic properties of the SDF-dECM patch (Fig. S1A). The dECM patches (*n* = 4 independent samples) exhibited a biphasic mechanical response with two distinct linear regions in their stress-strain curves. At strains < 12%, the patches showed a relatively low tangential modulus of 0.32 ± 0.05 MPa; beyond 12% strain, the material entered a pronounced strengthening phase, with the tangential modulus significantly increasing to 1.93 ± 0.21 MPa (Fig. S1B).

### Homing effect of endogenous BMMSCs induced by the SDF-dECM patch

**Fig. 4:**
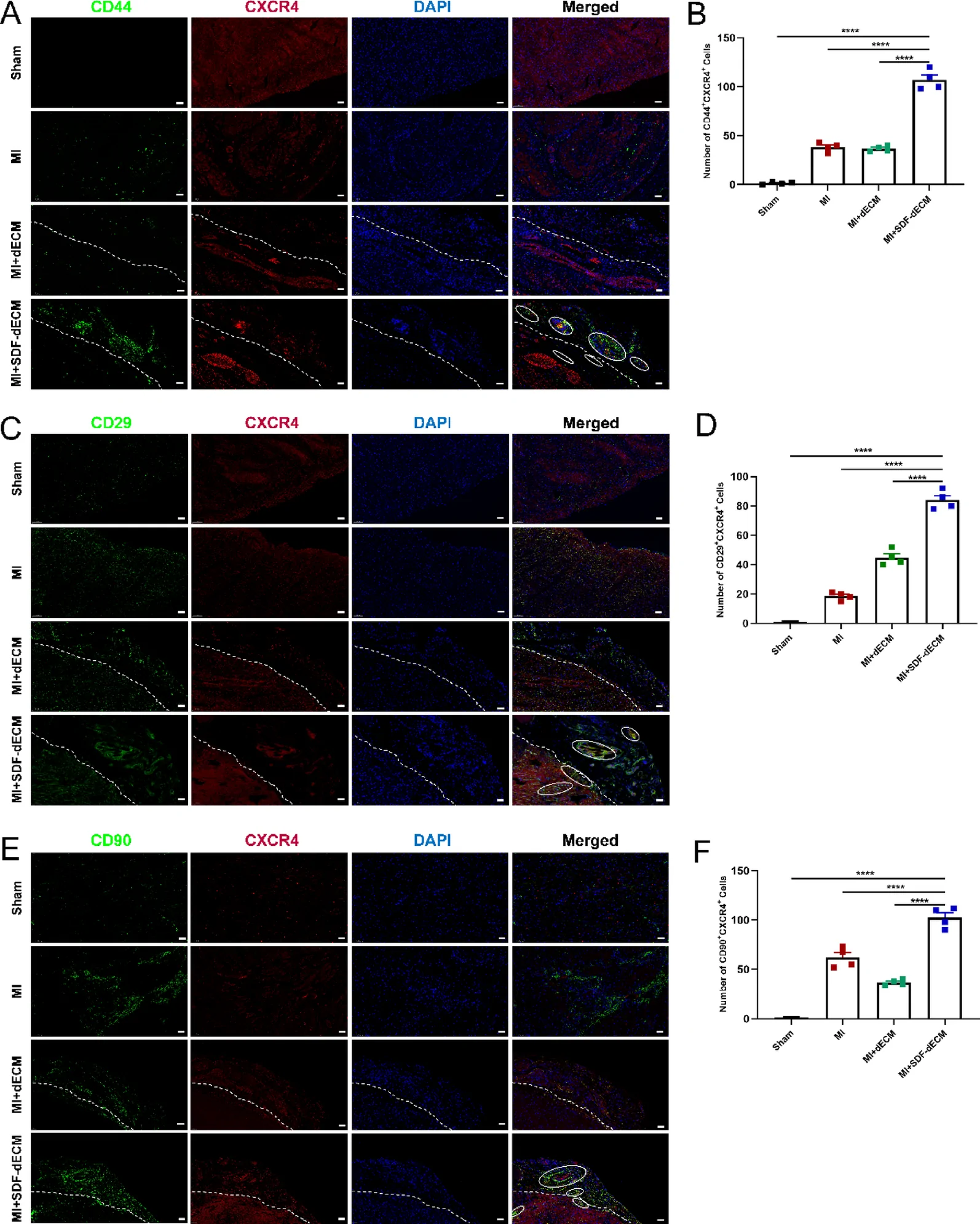
Immunofluorescence staining of BMMSCs markers in myocardium. **A** Representative immunofluorescence images of BMMSCs markers (CD44, green) and CXCR4 (red) along with nuclei counterstained using DAPI (blue), showing the localization of BMMSCs within the myocardium. **B** Quantitative analysis of CXCR4 and CD44 co-expressing cells. Bar graph shows the number of CXCR4^+^CD44^+^ cells. **C** Representative immunofluorescence images of BMMSCs markers (CD29, green) and CXCR4 (red) along with nuclei counterstained using DAPI (blue), showing the localization of BMMSCs within the myocardium. **D** Quantitative analysis of CXCR4 and CD29 co-expressing cells. Bar graph shows the number of CXCR4^+^CD29^+^ cells. **E** Representative immunofluorescence images of BMMSCs markers (CD90, green) and CXCR4 (red) along with nuclei counterstained using DAPI (blue), showing the localization of BMMSCs within the myocardium. **F** Quantitative analysis of CXCR4 and CD90 co-expressing cells. Bar graph shows the number of CXCR4^+^CD90^+^ cells. The patch is located in the upper right region, and the host myocardium is in the lower left region. The white dotted line indicates the boundary between them, while the cluster of BMMSCs on the patch is highlighted with white solid circles. Scale bar: 100 μm. Data are presented as mean ± SEM (*n* = 4 per group), ****p < 0.0001

Immunofluorescence staining was performed for BMMSCs surface markers (CD44, CD29, CD90) and CXCR4 in myocardial tissues from the four experimental groups (Fig. 4A, C, E). In representative images, BMMSCs clusters on the patch were highlighted with white solid circles to indicate their specific localization. Quantitative analysis showed that the MI + SDF-dECM group had significantly more CXCR4^+^CD44^+^ (Fig. 4B), CXCR4^+^CD29^+^ (Fig. 4D), and CXCR4^+^CD90^+^ (Fig. 4F) cells than all other groups (*p* < 0.0001). Notably, the patch (upper right region) integrated with the host myocardium (lower left region), with the boundary demarcated by a white dotted line.

### Promotion of microvascular regeneration and suppression of cardiomyocyte apoptosis by the SDF-dECM patch

**Fig. 5:**
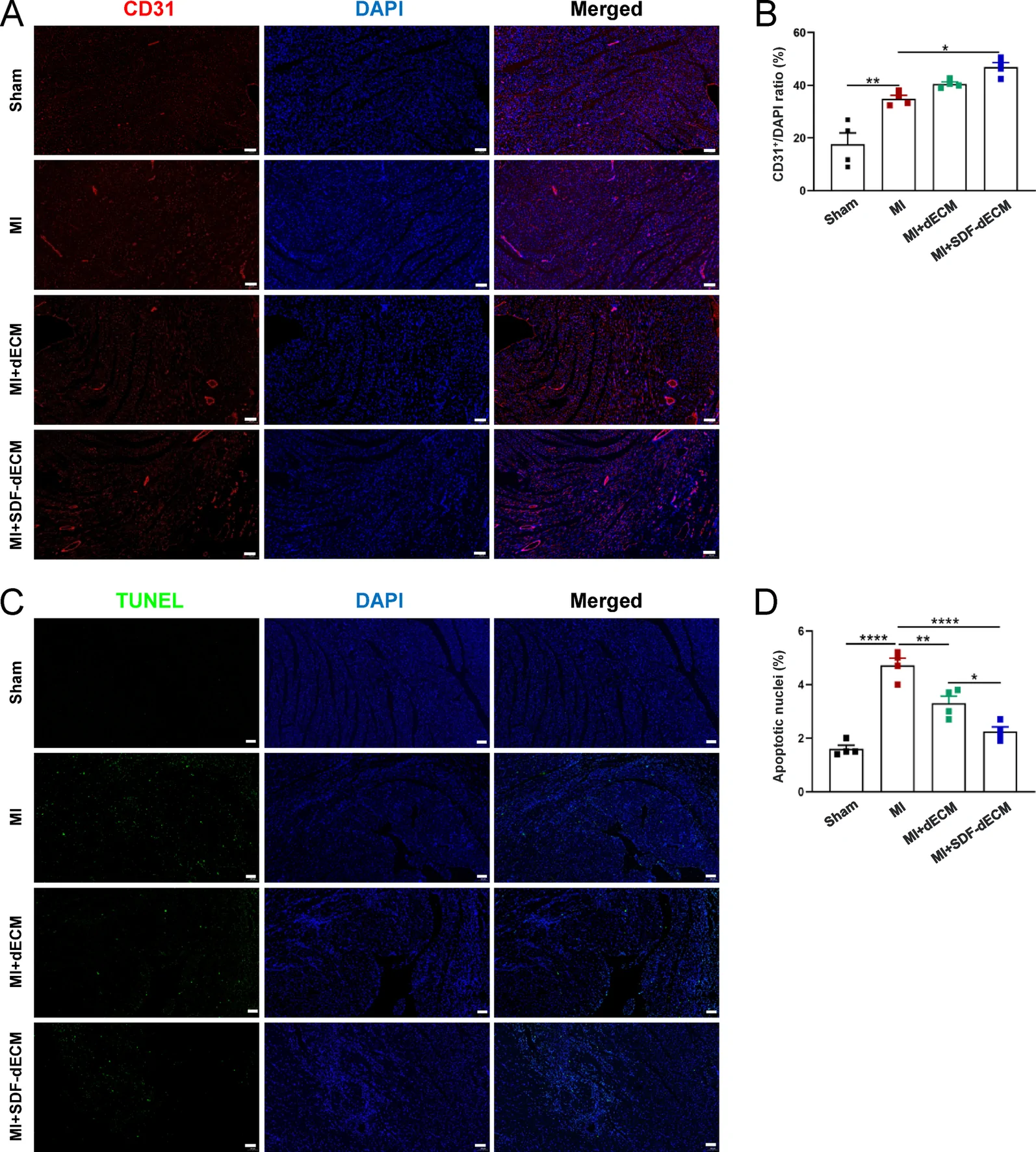
Vascular and apoptotic analysis in myocardium. **A** Representative immunofluorescence images showing CD31 (red)-positive endothelial cells along with DAPI-counterstained nuclei (blue) in myocardium, indicating blood vessel formation. Scale bar: 100 μm. **B** Quantification of CD31-positive endothelial cells (CD31^+^/DAPI ratio). Statistical significance: **p < 0.01, *p < 0.05 vs. control group. **C** TUNEL assay showing apoptotic cells (green, apoptotic cells) along with DAPI-counterstained nuclei (blue) in myocardium. Scale bar: 100 μm. **D** Quantification of apoptotic cardiomyocytes (TUNEL^+^/DAPI ratio). Significance levels: ****p < 0.0001, **p < 0.01, *p < 0.05 vs. Sham group

CD31 staining showed distinct peri-infarct neovascularization patterns, with the MI + SDF-dECM group exhibiting the highest microvascular density, confirming the patch’s enhanced angiogenic potential (Fig. 5A, B). TUNEL staining revealed minimal cardiomyocyte apoptosis in the Sham group, whereas the MI group showed significant apoptosis (*p* < 0.0001 vs. Sham group). Both the MI+dECM and MI + SDF-dECM groups had significantly reduced cardiomyocyte apoptosis compared to the MI group (*p* < 0.01), with the MI + SDF-dECM group providing superior protection relative to the MI+dECM group (*p* < 0.05) (Fig. 5C, D).

### Paracrine mechanism of BMMSCs for angiogenesis and anti-cardiomyocyte apoptosis

BMMSC-CM was collected from passage 3-5 BMMSCs at 80% confluence after 48-hour serum-free culture. For H9c2 treatment, cells were exposed to 50% BMMSC-CM under hypoxic conditions (1% O₂) for 24 h. ELISA analysis showed that VEGF and HGF concentrations in BMMSC-CM were significantly higher than those in the control (cell culture medium) and H9c2-CM groups (*p* < 0.0001) (Fig. S2A-B). In the BMMSC-CM group, CMECs progressively formed robust vascular structures, with total tubule length increasing significantly at 2, 4, and 6 h, whereas minimal network formation was observed in the control and H9c2-CM groups (Fig. S3A). Quantitative analysis confirmed that BMMSC-CM significantly increased the number of vascular tubes (Fig. S3B, *p* < 0.001) and total tubule length (Fig. S3C, *p* < 0.05) compared to control groups, highlighting the strong pro-angiogenic potential of BMMSCs.

The anti-apoptotic effects of BMMSC-CM were evaluated by subjecting H9c2 cardiomyocytes to hypoxia-ischemia (HI) followed by Hoechst 33,342 staining. The control group showed faint blue fluorescence and uniform morphology (minimal apoptosis), while HI-treated cells exhibited intense blue fluorescence, nuclear condensation, and a marked increase in apoptotic cells. In contrast, HI+BMMSC-CM-treated H9c2 cells displayed reduced fluorescence intensity and fewer apoptotic nuclei (Fig. S3D). Quantitative analysis revealed that HI-induced apoptosis was over twice that of the control group (*p* < 0.001), and BMMSC-CM treatment significantly reduced HI-induced apoptosis (*p* < 0.01 vs. HI alone group) (Fig. S3E).

### Mechanical supporting of the SDF-dECM patch

H&E and Masson Trichrome staining were performed on myocardial tissues and dECM from the Sham and MI groups. In the Sham group, myocardial fibers were well-aligned and densely packed without significant fibroplasia or inflammatory infiltration, whereas the MI group showed disorganized, loosely arranged myocardial fibers (Fig. S4A). Similarly, Sham group dECM exhibited a dense, well-organized collagen and elastic fiber network with intact structure, while MI group dECM had disrupted fiber organization and an irregular, fragmented grid pattern (Fig. S4B). These results confirmed that MI alters the three-dimensional collagen structure of dECM, leading to loss of mechanical integrity and support.

FEM simulations were conducted for cardiac diastole and systole. These simulations focused on the left ventricle (the primary MI-affected region in rats), and the model incorporated the SDF-dECM patch adhered to the infarct zone’s outer surface (based on experimental data; Fig. S5A-B). Left ventricular and infarct zone geometrical parameters are outlined in Fig. S5C; the ventricular wall was divided into three layers with distinct myocardial fiber orientations (Fig. S5D), and its mechanical properties were described using a fiber-reinforced hyperelastic constitutive model (Fig. S5E) [38]. The model also included sinoatrial node electrical stimulation propagation and subsequent myocardial contraction dynamics [35].

**Fig. 6:**
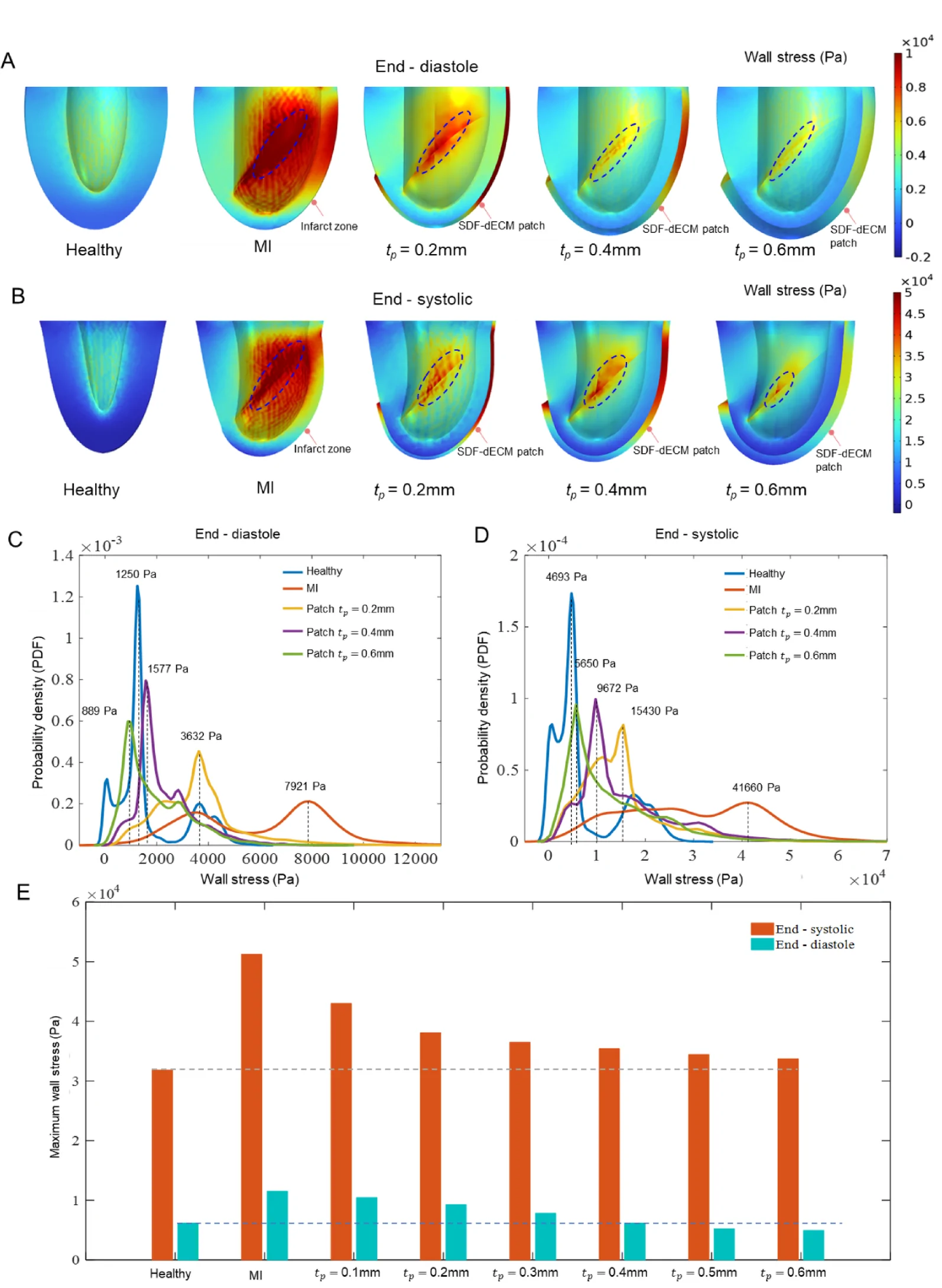
Mechanical support of SDF-dECM patch on WS. **A**, **B** WS distribution in the left ventricle during diastole and systole with and without the SDF-dECM patch, showing how the patch mitigates WS. Regions of WS concentration are highlighted by black dashed lines. **C**, **D** Probability density distributions of WS in the left ventricle during diastole and systole, demonstrate the patch’s effect on reducing WS variability. **E** Maximum WS values in the left ventricle during diastole and systole under different conditions, showing the mechanical support effect of the SDF-dECM patch

Simulation results (Fig. 6) compared WS in healthy, infarcted, and SDF-dECM patched ventricles. Post-MI, reduced infarct zone wall thickness increased WS, with significant stress concentrations at the healthy-infarct boundary during both diastole and systole (Fig. 6A, B). Increasing SDF-dECM patch thickness effectively reduced WS, with values approaching healthy myocardium at ≥ 0.4 mm thickness. WS distribution analysis showed that healthy ventricles had peak WS values of 1.25 MPa (diastole) and 4.69 MPa (systole) (Fig. 6C, D), which increased to 7.92 MPa and 41.7 MPa post-MI, respectively. While the SDF-dECM patch reduced these elevated stresses, excessively thick patches caused diastolic WS to fall below healthy levels (over-constraining cardiac motion). Additionally, maximum WS at the infarct boundary remained higher than healthy tissue for all patch thicknesses, with diminishing stress reduction beyond 0.4 mm (Fig. 6E). These findings indicate an optimal patch thickness of 0.4-0.6 mm balances WS reduction and normal cardiac function maintenance.

### Improvement of cardiac function in rats implanted with the SDF-dECM patch after MI

**Fig. 7:**
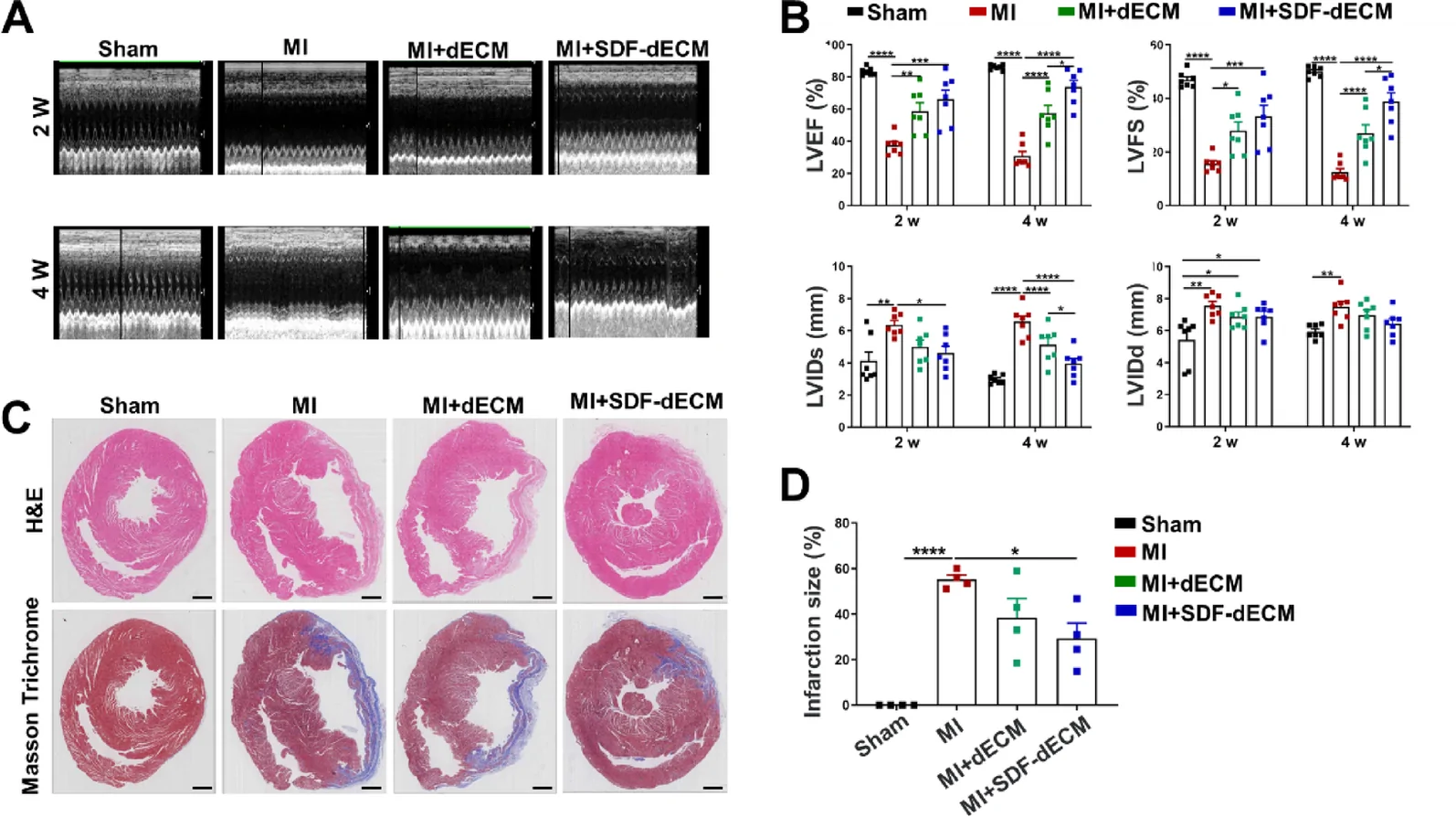
Echocardiographic and histological analysis. **A** Representative echocardiographic images at 2 and 4 weeks post-MI to track heart function. **B** Quantitative analysis of echocardiographic parameters at 2 and 4 weeks indicating changes in heart function. **C** H&E and Masson Trichrome staining of myocardial sections to assess tissue structure and fibrosis. Scale bar: 1 mm. **D** Quantitative assessment of infarction size across experimental groups. Statistical significance is denoted as *p < 0.05, **p < 0.01, ***p < 0.001, and ****p < 0.0001

Echocardiography at 2 and 4 weeks post-MI showed that at 2 weeks, LVEF and LVFS were significantly higher in the MI + SDF-dECM group than in the MI group (*p* < 0.001), with smaller LVIDs in the MI + SDF-dECM group (*p* < 0.05); LVIDd and LVIDs were larger in the MI group than in the Sham group (*p* < 0.01). At 4 weeks, both patched groups had improved LVEF and LVFS vs. the MI group (*p* < 0.0001), with the MI + SDF-dECM group showing higher LVEF, LVFS and smaller LVIDs than the MI+dECM group (*p* < 0.05) (Fig. 7A, B). H&E and Masson Trichrome staining revealed thicker left ventricular walls, reduced fibrosis, and better viable myocardial preservation in both patched groups vs. the MI group, with the MI + SDF-dECM group performing superiorly (Fig. 7C). Quantitative infarct analysis showed the largest area in the MI group (*p* < 0.0001); the MI + SDF-dECM group had a significantly smaller infarct area (*p* < 0.05), while the MI+dECM group had a non-significant reduction (Fig. 7D).

## Discussion

Tissue engineering exhibits tremendous potential for applications in tissue regeneration. Although natural scaffolds and synthetic polymer-based materials have undergone rapid development and widespread utilization, their application in myocardial repair remains limited by persistent challenges, such as immunogenicity and insufficient recapitulation of the native myocardial microenvironment, architectural structure, and physicochemical properties. dECM scaffolds effectively eliminate immunogenic cellular components while preserving physiological structures and native biomolecular compositions, thus providing a favorable microenvironment for tissue repair and regeneration. In the present study, we pioneered the integration of mechanically robust dECM scaffolds with SDF-1, a potent chemotactic factor, and demonstrated its ability to chemoattract endogenous BMMSCs and reduce infarct WS, thereby attenuating adverse myocardial remodeling and enhancing cardiac function in vivo.

In vitro co-culture experiments confirmed the excellent cytocompatibility of porcine-derived dECM patches with xenogeneic rat BMMSCs and H9c2 cardiomyoblasts. GFP-labeled BMMSCs showed vigorous proliferation and effective integration within the three-dimensional porous structure of the dECM scaffolds, underscoring their capacity to promote favorable cell-scaffold interactions and support cellular functions during recellularization [39]. Although our in vitro studies demonstrated excellent cytocompatibility, a comprehensive assessment of the immunogenicity induced by xenogeneic dECM components was not conducted in the present study. Future studies should include detailed analysis of host immune response, including lymphocyte infiltration and cytokine production profiles.

Following MI, elevated SDF-1 levels in infarcted regions promote stem cell homing to injury sites via binding to the CXCR4 chemokine receptor [37, 40]. Although the molecular mechanisms underlying the migration and homing of MSCs remain to be fully elucidated [41], accumulating evidence has implicated CXCR4 as a pivotal mediator [42]. While MSCs express functionally active CXCR4 [40], endogenous SDF-1 levels decline progressively following MI [43]. Transient upregulation of SDF-1 within hours after MI enhances cardiac-directed stem cell recruitment [44]. However, this chemotactic response is significantly diminished between 72 and 96 h after MI [43]. Notably, our SDF-dECM patches enabled sustained SDF-1 release without attenuation for 28 days, thereby prolonging both chemokine release duration and the therapeutic window for endogenous BMMSCs homing to infarcted myocardial regions.

The SDF-1/CXCR4 axis represents the principal signaling pathway governing stem cell mobilization and migration, with SDF-1 acting as the master regulator of CXCR4^+^ stem cell trafficking to injured tissues. A concentration gradient of SDF-1 drives the directional migration of CXCR4^+^ cells [45], a mechanism conserved across species and injury models [46, 47]. For instance, increased SDF-1 levels at fracture sites recruit MSCs to facilitate osteogenesis [48], whereas upregulation of ovarian SDF-1 following chemotherapy directs MSC homing to support tissue repair [49]. In myocardial ischemia, localized hydrogel-mediated delivery of SDF-1 enhances endogenous BMMSCs recruitment, angiogenesis, and cardiomyocyte survival by restoring the ischemic microenvironments [48]. Acting as an upstream signaling hub, SDF-1/CXCR4 ligation activates multiple downstream pathways, including Akt, ERK1/2, focal adhesion kinase, and p38 [50], thereby orchestrating diverse biological responses such as chemotaxis and cell adhesion [51].

Mounting evidence indicates that the therapeutic benefits of BMMSCs in cardiac repair are largely mediated by paracrine signaling, which promotes angiogenesis and inhibits cardiomyocyte apoptosis [52]. BMMSCs secrete diverse bioactive factors, including exosomes, dECM components, growth factors, cytokines, chemokines, and microRNAs [53, 54]. These factors enable BMMSCs to interact with adjacent cells and regulate critical processes such as cell survival, tissue regeneration, neovascularization, anti-inflammatory responses, and fibrosis inhibition [55]. Notably, VEGF and HGF are key paracrine mediators secreted by BMMSCs, supporting cell survival and proliferation, modulating immune responses, regulating cardiac remodeling, enhancing angiogenesis, and activating endogenous repair pathways [52, 56, 57]. Given their paracrine activity, BMMSCs hold great therapeutic potential for targeting the complex pathophysiology of IHD. Their capacity to induce vascularization and pr otect cardiomyocytes from apoptosis further establishes BMMSCs-based therapies as a promising strategy for regenerative cardiac medicine [52].

The therapeutic efficacy of an implanted SDF-dECM patches is closely linked to their mechanical properties, which mediate dynamic crosstalk with host cells and drive in vivo mechanical remodeling. Our observations of altered collagen fiber morphology and host cell recruitment within the implant area confirm such mechanical- biological interplay, consistent with prior work by Sarig et al. [58]. This aligns with our overarching strategy of designing bioactive, mechanically compatible dECM scaffolds, supported by Krishnamoorthi et al. [33], who emphasized the critical role of robust fabrication techniques in directing scaffold-mediated biological outcomes.

Myocardial ischemia induces irreversible cardiomyocyte loss, fibrosis, scarring, and ventricular wall thinning, collectively elevating WS. Guided by Laplace’s law [59], myocardial patches mitigate ventricular remodeling via mechanical reinforcement by augmenting infarct zone thickness to reduce WS. Our finite element simulations systematically evaluated left ventricular WS distribution under varying patch thicknesses, coverage areas, and pre-stretch strains, revealing pronounced WS in infarcted regions and stress concentration at infarct-borderzone interfaces. Patches effectively attenuated both infarct-related WS and borderzone stress concentration, with increased thickness, area, and pre-stretch strain synergistically reducing WS—pre-stretch exhibited superior efficacy at optimal thickness/area combinations. However, excessive parameter escalation reduced left ventricular end-diastolic volume (LVEDV), potentially impairing cardiac output, underscoring the need for balanced optimization of patch parameters to harmonize WS reduction, ventricular compliance, and hemodynamic performance.

Mechanical characterization demonstrated that porcine myocardial SDF-dECM patches exhibit minimal hysteresis and strain-stiffening elastic behavior under cyclic loading— a critical property for withstanding dynamic cardiac physiological forces without mechanical failure or plastic deformation, consistent with Bronshtein et al. [60]. Au-Yeung et al. [16] further confirmed that recellularization modulates SDF-dECM mechanical compliance toward a native-like state, supporting our design of SDF-dECM patches as scaffolds for host cell recruitment and mechanical remodeling. 

### Study limitations

Several limitations should be considered when interpreting our results. First, while in vitro experiments with rat BMMSCs showed promise, these findings are indicative rather than conclusive for the in vivo mechanisms. Infarct-recruited cells in vivo may not be exclusively bone marrow-derived, and the surface markers used (CD29, CD44, CD90) are expressed across multiple mesenchymal lineages, limiting definitive cell population characterization. Second, dECM patch characterization relied primarily on qualitative histological staining. We acknowledge the established standards for decellularized biomaterials, which include quantitative residual DNA content analysis [61]. Third, although SDF-1 supplementation significantly improved cardiac function parameters, the clinical relevance of this modest benefit requires validation in larger preclinical studies with extended follow-up. Additionally, our finite element modeling provides theoretical insights but relies on assumptions that may not fully capture the complex in vivo mechanical environment. Finally, while histological staining (H&E, DAPI) and electron microscopy confirmed effective decellularization, we did not perform quantitative biochemical assays as outlined in established dECM guidelines [61].

## Conclusion

The SDF-dECM patch exhibits excellent biocompatibility and enables stable, sustained release of SDF-1, which significantly enhances the recruitment of endogenous BMMSCs to the infarcted myocardium via the SDF-1/CXCR4 axis. This approach promotes microvascular regeneration and reduces cardiomyocyte apoptosis in the infarcted region through paracrine signaling from the recruited BMMSCs. Additionally, FEM simulations demonstrate that the SDF-dECM patch significantly improves LVEF and overall cardiac function in rats with MI. This dual-functional strategy, integrating biological activity with biomechanical support, represents a promising therapeutic approach for IHD.

## Supplementary Information

Below is the link to the electronic supplementary material.

Supplementary Material 1.

## Data Availability

No datasets were generated or analysed during the current study.

## Abbreviations

IHD: Ischemic heart disease
HF: Heart failure
ECM: Extracellular matrix
dECM: Decellularized extracellular matrix
MI: Myocardial infarction
WS: Wall stress
MSC: Mesenchymal stem cell
SDF-1: Stromal cell-derived factor-1
CXCR4: C-X-C chemokine receptor type 4
BMMSCs: Bone marrow mesenchymal stem cells
H&E: Hematoxylin and eosin
DAPI: 4’,6-diamidino-2-phenylindole
SEM: Scanning electron microscopy
CCK-8: Cell counting kit-8
ELISA: Enzyme-linked immunosorbent assay
CMECs: Cardiac microvascular endothelial cells
LAD: Left anterior descending
ECG: Electrocardiography
FEM: Finite element method
VEGF: Vascular endothelial growth factor
HGF: Hepatocyte growth factor
BMMSC-CM: BMMSCs-conditioned medium
H9c2-CM: H9c2-conditioned medium
LVEF: Left ventricular ejection fraction
LVFS: Left ventricular fractional shortening
