# Supplementary material for: SDF-1 loaded dECM patch improves cardiac function in rats after myocardial ischemia
Source: Stem Cell Res Ther. 2026 Jul 18;17:298. doi: 10.1186/s13287-026-05188-x (PMC13545792; doi:10.1186/s13287-026-05188-x)
Supplement: Supplementary file 1 [file 13287_2026_5188_MOESM1_ESM.docx]

**SDF-1 loaded dECM patch improves cardiac function in rats after** **myocardial ischemia**

Jing Zhang^1*^, Wanqing Lin^2^, Qian Li^3^, Shiyu Cui^3^, Xiang Bu^1^, Xin Jiang^1*^, Aiqun Ma^3*^, Tingzhong Wang^3*^

^1^ Department of Cardiology, Shaanxi Provincial People’s Hospital, Xi’an, Shaanxi, 710068, China.

^2^ Laboratory for Multiscale Mechanics and Medical Science, Xi'an Jiaotong University, Xi’an, Shaanxi, 710049, China.

^3^ Department of Cardiology, The First Affiliated Hospital of Xi’an Jiaotong University, Xi’an, Shaanxi 710061, China.

*Corresponding authors:

Jing Zhang ([zjxjtu@spph-sx.ac.cn](mailto:zjxjtu@spph-sx.ac.cn)), Xin Jiang ([Jiangxinwind@126.com](mailto:Jiangxinwind@126.com)), Aiqun Ma ([aiqun.ma@xjtu.edu.cn](mailto:aiqun.ma@xjtu.edu.cn)), Tingzhong Wang ([tingzhong.wang@xjtu.edu.cn](mailto:tingzhong.wang@xjtu.edu.cn)).

**Figure S1.** Mechanical properties of dECM. A) Illustration of the deformation behavior of the dECM during tensile testing. B) Stress-strain curves for the three tested samples, showing two distinct linear regions with moduli of 0.32 ± 0.05 MPa and 1.93± 0.21 MPa, respectively.

**Figure S2.** Detection of VEGF and HGF levels in conditioned medium. A) Statistical analysis of VEGF concentration in the conditioned medium. B) Statistical analysis of HGF concentration in the conditioned medium, *****p <* 0.0001.

**Figure S3**. BMMSC-CM promoted angiogenesis and reduced cardiomyocyte apoptosis. A) Representative images of CMECs tube formation at 2, 4, and 6 hours post-treatment under different conditions. Scale bar: 100 μm. B) Quantitative analysis of the total number of tube nodes formed by CMECs treated with different conditioned media. C) Quantitative analysis of the total branch length of tubes formed by CMECs treated with different conditioned media. Statistical significance is denoted as **p* < 0.05, ***p <* 0.01, ****p <* 0.001, and *****p <* 0.0001. D) Hoechst 33342 staining of H9c2 cardiomyocytes to assess apoptosis. Scale bar: 150 μm. E) Quantitative analysis of the apoptosis rate of H9c2 cells. Statistical significance is denoted as **p <* 0.05, ***p <* 0.01, and ****p <* 0.001.

**Figure S4.** Histochemical staining of rat myocardium and dECM. A) H&E and Masson Trichrome staining of myocardial tissue in the Sham and MI groups, scale bar: 2 mm. B) H&E and Masson Trichrome staining of myocardial dECM in the Sham and MI groups, scale bar: 100 μm.

**Figure S5**. Cardiac infarction and finite element models. A) Gross specimen of the myocardium and H&E staining of transverse sections following myocardial infarction. B) Schematic representation of the cardiac patch model. C) Finite element model with corresponding dimensions. D) Fiber orientation of the left ventricle. E) Boundary and initial conditions applied to the model.

**Table S1** Material parameters used in FEM model

| Parameters | Value | Description | |
| --- | --- | --- | --- |
| $a_{i}$ | 2.280 kPa | | Isotropic Material Properties |
| $b_{i}$ | 9.726 | | Isotropic Material Properties |
| $a_{f}$ | 1.685 kPa | | Cardiac Fiber Material Properties |
| $b_{f}$ | 15.779 | | Cardiac Fiber Material Properties |
| $\rho_{s}$ | 1370$\text{ }\text{kg/m}\text{3}$ | | Myocardial Density |
| $C_{1}$ | 0 | | Shear Modulus Parameter of the Kinematic Mesh |
| $C_{2}$ | 1 | | Nonlinear Mesh Stiffness Parameter of the Kinematic Mesh |
